# Insights into the medical management of gastrointestinal stromal tumours: lessons learnt from a dedicated gastrointestinal stromal tumour clinic in North India

**DOI:** 10.3332/ecancer.2023.1497

**Published:** 2023-01-16

**Authors:** Annie Kanchan Baa, Sameer Rastogi, Sanal Fernandes, Shakti Shrivastava, Rajni Yadav, Adarsh Barwad, Shamim A Shamim, Nihar Ranjan Dash

**Affiliations:** 1 Department of Medical Oncology, Dr B R Ambedkar Institute Rotary Cancer Hospital, All India Institute of Medical Sciences, New Delhi 110029, India; 2 Department of Pathology, All India Institute of Medical Sciences, New Delhi 110029, India; 3 Department of Nuclear Medicine, All India Institute of Medical Sciences, New Delhi 110029, India; 4 Department of Gastrointestinal Surgery, All India Institute of Medical Sciences, New Delhi 110029, India

**Keywords:** molecular testing, mutation, GIST, tyrosine kinase inhibitors (TKIs)

## Abstract

**Background:**

The advent of molecular driver alterations has brought in a revolutionary transformation in the treatment landscape of gastrointestinal stromal tumour (GIST). However, there is a paucity of data regarding mutational testing prevalence and associated outcomes from India.

**Methods:**

It was a retrospective study. We reviewed the case records of all patients diagnosed with GIST in a tertiary care centre from 2015 to 2021. The clinicopathological, mutational analysis and treatment plans were recorded. The study cohort was characterised by descriptive statistics.

**Results:**

Our study included 120 patients with a median age of 53 years (range: 28–77), with a male preponderance of 2:1. The most common site of the primary was the stomach (50%), followed by the small intestine (37%), with 55.8% of the patients having disseminated disease at presentation with a predominance of liver metastasis (67%). The prevalence of mutational analysis among patients prior to referral was 4%. 60.8% of the patients at our clinic had mutational analysis performed, and unavailability of analysis in the rest was due to financial constraints (12.5%), exhaustion of tissue (7.5%), reluctance to repeat biopsy (4.1%) and low-risk patients. We report c-kit in the majority (52%), platelet-derived growth factor receptor (PDGFR) in 19.2% and wild type in 16.4% along with the rarer subtypes: succinate dehydrogenase (SDH)-deficient GIST in 10.9% and Neurotrophic tyrosine receptor kinase (NTRK) fusion in 1.3%. Four of the eight SDH-deficient GIST patients had germline mutations (50%). The knowledge of driver mutations led to a change of treatment in 39.7% (29/73), i.e. stoppage of tyrosine kinase inhibitor (TKI) in 3, switch of TKI in 23, increase in TKI dose in 2 and upfront surgery in 1. The most common change was the use of sunitinib and regorafenib in patients with SDH-deficient GIST.

**Conclusion:**

Our study is one of the largest comprehensive series describing the clinical and mutational profile of GIST from India. The mutation testing rates at primary care centres continue to be low. Despite the hurdles, a large percentage of our patients underwent molecular testing, aiding in therapeutic decision-making.

## Introduction

Gastrointestinal stromal tumours (GISTs) are the common mesenchymal tumours encountered in the alimentary tract, having an incidence of 1:100,000 per year [1]. The discovery of KIT mutation brought about a revolution in the management of GIST [2]. Targeted therapy has now become the cornerstone, which was solely governed by surgery early on.

Localised GIST has treatment guided by various nomograms and depends upon the site, mitotic count, size and mutation analysis [3, 4]. Adjuvant imatinib is recommended in high risk for 3 years as established in the long-term analysis of the SSG VIII/AIO adjuvant trial. The 5-year and 10-year overall survival (OS) was 92% versus 85% and 79% versus 65.3%, respectively (3 years versus 1 year of adjuvant imatinib) [5].

Systemic therapy remains the mainstay of treatment in patients with advanced GIST. Earlier on, in the metastatic setting, Phase 3 SWOG Intergroup Trial S0033 had shown better progression-free survival and OS rates in patients having exon 11 mutation receiving imatinib. Subsequently, approval for sunitinib in the second line and regorafenib in the third line, respectively, was granted [6]. Novel tyrosine kinase inhibitors (TKIs) have opened a new arena: avapritinib has been accredited for PDGFRA D842V in upfront and metastatic settings, while ripretinib has strengthened the armamentarium in the fourth line. SDH-deficient and wild-type GISTs have shown decent response rates (RRs) with sunitinib and regorafenib as compared to imatinib [7–9]. Thus, molecular subtyping is instrumental in guiding the right choice of TKIs.

While the research in the developed world is moving towards precision medicine, liquid biopsy, elaborate genomic classification and immunotherapy, the developing world has altogether different research questions [6, 9, 10]. Currently, mutation testing is advised for all patients of GIST in adjuvant and metastatic settings. Exceptions to this norm are patients with low-risk GIST (decided by various nomograms) who undergo complete resection. There are numerous challenges that developing countries are facing as reflected by sparse data, fewer reports of mutation testing, lack of GIST new trials, the absence of reporting of wild-type GIST, difficulty in procuring newer drugs, few support groups, lack of dedicated centres and lack of collaboration. Indian data describing and analysing the mutations is sparse [11–14]. Minhas *et al* [15] and Cyriac *et al* [16] described a cohort of 31 and 24 patients, respectively, wherein c-kit was the most common mutation seen. SDH deficiency, wild type and PDGFRA D842V molecular alterations have been incorporated in the guidelines to better understand the extensive clonal heterogeneity observed in patients with GIST. A larger comprehensive cohort addressing the mutational profile including experience with the newer agents (avapritinib and ripretinib: approved in 2020) is warranted. We highlight the lessons learnt from a tertiary care centre with a dedicated GIST/sarcoma clinic. The use of novel therapies, guidance by molecular testing (including wild-type GIST/SDH mutations) and the clinicoradiological outcomes have been elaborated in our current study.

## Materials and methods

Ethical approval was taken prior to the commencement of the study via IEC-601/03.09.2021, RP-24/2021. We retrospectively reviewed case files of all patients diagnosed with GIST from 2015 to 2021 at our tertiary centre. Clinicopathological details, mutational analysis along with treatment strategies were collected. We updated the recent follow-up telephonically. Statistical Package for the Social Sciences software was used for data analysis (IBM SPSS v.26). The study cohort was characterised by descriptive statistics. In 2015, the GIST and Sarcoma clinic was started in collaboration with our pathology, radiology, nuclear medicine, gastrointestinal surgery and radiation oncology colleagues. Multi-disciplinary discussion regarding management entailed an essential element before any proceedings. CD117, DOG1 positivity on immunohistochemistry (IHC) mandated the histopathological diagnosis of GIST. SDH deficiency was based on IHC, with endothelial cells of blood vessels used as an internal control to compare the SDH loss in tumour cells. Somatic and germline SDH mutation testing was performed in patients with SDH-deficient GIST. Mutation analysis was performed by polymerase chain reaction using DNA extracted from formalin fixed paraffin embedded tissue. Newer TKIs were procured on a compassionate basis: avapritinib with the help of blueprint and WEPclinic and ripretinib via Clinigen Group. Risk stratification for adjuvant TKI was done using the Armed Forces Institute of Pathology (AFIP) criteria [4, 17].

## Results

**Figure 1.**
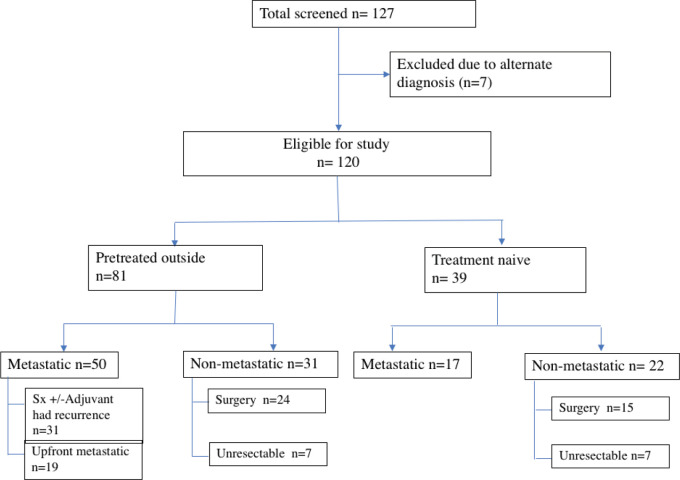
Flowchart of the study.

A total of 127 patients were registered for GIST. However, seven patients were excluded as the final diagnosis differed from the preliminary diagnosis of GIST. The differentials creating the dilemma were malignant peripheral nerve sheath tumour, synovial sarcoma, leiomyosarcoma and carcinoma stomach/adenoma. Subsequently, 120 patients were analysed and characterised as per the recorded data (Figure 1).

The proportion of patients being referred after treatment at a primary centre was higher, accounting for 67.5% (81/120) of the cohort. 61.7% (50/81) of patients were metastatic at presentation to our centre, out of which 38% (19/50) had disseminated disease at the time of diagnosis outside. They had received one line of medical therapy, i.e. TKI and mutation analysis was available only for four patients (4/81; 4.9%).

The baseline features of the cohort have been summarised in Table 1. The median age of the cohort was 53 years (range: 20–79 years) with a male preponderance of 67.5%. The common symptoms at presentation were abdominal discomfort (61/120), malena (23/120), generalised weakness (14/120), early satiety (6/120) and constipation (6/120). Two patients had haematemesis upfront. Incidental detection was observed in four patients when ultrasonography was done at a routine check-up.

**Figure 2.**
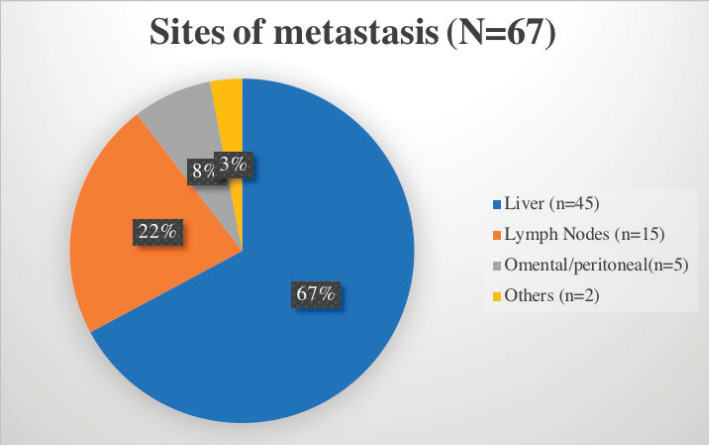
Common sites of metastasis in GIST encountered in our cohort.

The stomach (50%) was the most common site of disease occurrence at diagnosis followed by the small intestine (35%). Omental and peritoneal lesions were also seen in a few (4.1%). Rare sites of GIST were also seen such as anorectum (3.3%). The frequently encountered site of metastasis was liver (67%) followed by lymph nodes (Figure 2).

In the non-metastatic cohort, 73.5% (39/53) underwent primary resection, while 26.4% (14/53) were unresectable and were initiated on neoadjuvant TKI. Post-surgery, the patients were classified into high (26/39), intermediate (4/39) and low risk (9/39), based on AFIP criteria (Table 2).

Fourteen patients (26.45%) were initiated on neoadjuvant TKI in view of unresectability at presentation. All were started on imatinib (duration: 3 months – four patients, 6 months – seven patients). Eleven patients underwent a resection post neoadjuvant therapy (three were lost to follow-up). Post-surgery, adjuvant TKI was administered in six patients based on the risk stratification as per AFIP criteria.

**Figure 3.**
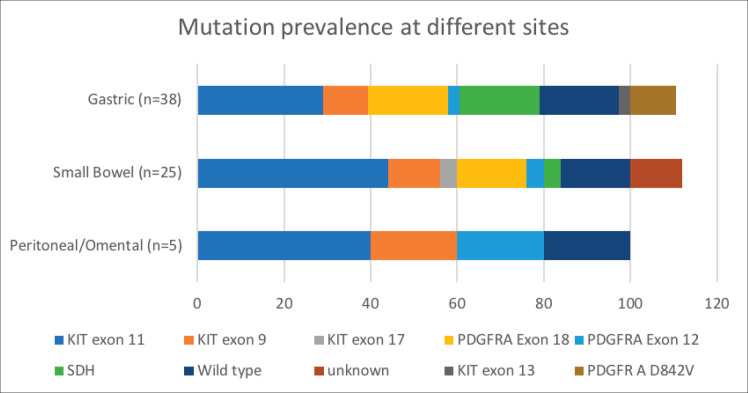
Mutation prevalence based on site of origin of GIST. *Data available for 38 out of 60 patients with gastric GIST; 25 out of 41 patients in patients with small bowel GIST.

Mutational analysis was available for 60.8% (73/120) of the cohort. The most commonly encountered mutation was c-kit (52%; 38/73) at exon 11 followed by exon 9. PDGFR exon 18 accounted for 19.2% (14/73), with D842V molecular alteration detected in four patients. Wild type was seen in 12 (16.4%) and SDH deficiency was seen in 8 patients (10.9%) while an NTRK translocation was observed in 1 patient. Germline testing was done for all SDH-deficient patients, except but one. Four patients were found to harbour SDH mutation. The details are listed in Table 3. The prevalence of specific mutation according to the origin of GIST is elaborated in Figure 3. C-kit exon 11 was the predominant mutation seen across all the primary sites. PDGFR exon 18-D842V substitution (4/4) and SDH deficiency (7/8) were documented in stomach.

We also report two patients with genetic syndromes associated with GIST. Carney’s triad was seen in a 48-year-old male having gastric GIST harbouring SDH mutation. Neurofibromatosis-type 1 (NF1) was diagnosed in a 29-year-old lady with wild type (c-kit/PDGFR negative and SDH retained) gastric GIST having café-au lait spots with her son also reporting similar macular skin lesions. NF1 mutation was detected in exon 45, which was heterozygous and dominant.

The patients deemed low-risk as per AFIP criteria (9/39) were excluded from molecular testing and kept on observation. However, a strong clinical suspicion prompted us to get status checked, in view of the therapeutic implications and familial genetic testing for one such patient. Failure to achieve results in the rest of the patients (39/120) was mainly due to exhaustion of tissue (*n* = 9), reluctance for a repeat biopsy (*n* = 5), financial constraints (*n* = 15), patients’ hesitancy (*n* = 6) and others (*n* = 4).

Imatinib (400 mg daily) was given for a period of 3 years for high-risk patients and lifelong in case of rupture. The non-imatinib sensitive mutations (wild-type (*n* = 1)/SDH-deficient subtypes (*n* = 1)) were offered sunitinib. The low-risk cohort (*n* = 9) was kept on observation irrespective of the molecular alteration and imatinib was stopped for those who had it initiated from outside (three out of nine).

The different TKIs used in metastatic setting with their RRs and adverse effects have been detailed in Table 3. Imatinib was employed in 83.5% (56/67) with a RR of 46.4%. It was tolerated well with no dose modification. Sunitinib at a dose of 37.5 mg daily was used in 15 patients’ after progression on imatinib, with dose reduction warranted in at least three patients. Regorafenib, due to the toxicity profile, was utilised at a lower dose of 80 mg (recommended dosing 160 mg) in the second line (two patients) and the third line (seven patients) [18].

We also report the use of novel agents procured on compassionate grounds. Avapritinib was used in the fourth line for five patients with PDGFR exon 18 in a dose of 300 mg once daily, while two patients received it upfront, owing to PDGFRA D842V mutation. Ripretinib 150 mg once daily was used in the fourth line for three patients. Dose escalation to 300 mg was made in one patient after disease progression with 150 mg of ripretinib, based on the study by George *et al* [19]. Overall mutational analysis changed the treatment course in 39.7% (29/73) of the patients undergoing testing (Table 4). The median follow-up for patients with metastatic disease at presentation was 20 months (range: 15–24 months). The 2-year OS of these patients as per their mutations is reported in Table 5.

## Discussion

GISTs are heterogeneous in terms of molecular signature and differ in their natural history and management [20]. This retrospective analysis is the largest study from India elaborating on the diversity appreciated in previous datasets across the world.

The median age at diagnosis is reported in a range of 65–69 years with a slightly higher male preponderance [6, 21]. Our cohort had a comparatively younger median age of 53 years at presentation while demonstrating a male-to-female ratio of 2:1. The most common site of origin reported is the stomach (55.6%) followed by the small intestine (31.8%) while uncommon sites are colorectum (6%), oesophagus (0.7%) and omentum/mesentery [15, 20, 21]. We report similar figures, with 50% and 35% occurrence seen in the stomach and small intestine, respectively, while rarer sites like anorectal GIST were seen in 3.3%.

67.5% (81/120) of the patients were treated outside before presenting to our clinic, with only 4.9% (4/81) having mutational analysis. This echoes the data from Life Raft Group patient registry, showing poor testing rates in GIST (6%) in 2010, which has now increased to 57% [22]. The PALGA study also reports that 33.9% of the patients post-resection had their mutations tested [23]. Emphasis on recognising the various molecular driver alterations has been lacking, despite the ESMO guidelines, which have been reiterated in the current study.

Our study highlights this less explored arena of GIST in the Indian subcontinent [15, 16, 24]. Seventy-three patients (65%) successfully underwent mutational analysis despite the hurdles at the pathological and financial levels (Table 1). We report a 52% (38/73) prevalence of C-KIT mutation, with alteration in exon 11 being the most common (68.4%; 26/38). This is lower, compared to 70%–80% of C-KIT prevalence which is reported in literature [25, 26]. PDGFRA gene was mutated in 19.2%, with exon 18 D842V substitution seen in 40% (4/10) (Table 1, Figure 3). The increased small bowel origin (35%) of PDGFR mutation is seen in our study. These numbers are higher in contrast with the described range of prevalence of 2%–14% along with predominant gastric origin [27].

Wild-type and SDH-deficient GISTs were seen in 16.4% and 10.9%, respectively. There has been only one case report of SDH-deficient GIST described from India to this date, which had been reported from our GIST clinic [28]. The associated genetic syndromes (Carney triad and NF1) have also been identified and elaborated earlier. SDH-deficient GIST is commonly reported in females (70%) with predilection in the stomach [29]. Our cohort also mirrored the site of occurrence in stomach (87.5%) but showed similar incidence in males (4/8) and females (4/8). SDH deficiency may either be due to mutation in the SDH family gene or through epigenetic silencing [29, 30]. Thus, it is important to identify the germline mutations as the family members need to be screened and kept on recommended follow-up. Also, trials to assess therapeutic modalities in wild-type GIST represent a large unmet need in Low middle income countries (LMIC) regions.

The percentage of patients with molecular analysis has thereby improved tremendously (4.9%–67.5%). This is a worthwhile achievement for our tertiary centre, as we compare our percentage with the latest data by Montoya *et al* [22]. The mutational analysis was instrumental in tailoring the treatment and management of GIST in approximately 40% of those who underwent testing (Table 6). Also, the confirmation of imatinib-sensitive mutations reassured the ongoing optimum therapy. We could also incorporate the novel agents under the aegis of identification of driver mutations. Verma *et al* [31] have illustrated the experience with avapritinib procured on a compassionate basis at our centre. Thus, early mutational analysis helps in personalising the use of TKI, thereby improving the prognosis and preventing the possible unwanted adverse effects by using the right drug. The incorporation of mutation testing at the onset has also proven to be more cost-effective than empirical imatinib in a few studies [32, 33]. However, similar studies in our population are warranted to establish the financial benefit. The high cost, difficulty in the procurement of newer drugs and lack of clinical trials make it a heaping task in a developing country leading to increased treatment discontinuation rates.

Our retrospective analysis had its own limitations. Firstly, due to the short follow-up periods, long-term outcomes and toxicities could not be described in our study. The detailed survival analysis could not be done owing to heterogeneous data. While the developed world is focusing on precision medicine and designing trials with newer TKIs and immunotherapy, we still struggle to get the mutational profile and deliver therapy [9, 34, 35].

## Conclusion

Our study is one of the largest series describing the driver mutations of GIST from India. The prevalence of mutation testing among patients with GIST continues to be low at the level of primary care centres. Mutational analysis testing helped us personalise treatment patterns in a significant proportion of patients attending our GIST clinic. This highlights the importance of mutational analysis for therapeutic decision-making, impacting the prognosis of patients as well as the family. Novel TKIs could be incorporated into our armamentarium of management of GIST. The geographical variations can be understood better if dedicated support groups and collaborations work synchronously, as entities continue to be ‘rare’ if active testing is lacking.

## Authors’ contributions

Study concept and design: S Rastogi and A K Baa. Acquisition, analysis and interpretation of data: S Rastogi, A K Baa, S Shrivastava, S Fernandes. Drafting of the manuscript: S Rastogi and A K Baa. Critical revision of the manuscript: all authors. All authors read and approved the final manuscript.

## Funding and conflicts of interest

The authors have no relevant affiliations or financial involvement with any organisation or entity with a financial interest in or financial conflict with the subject matter or materials discussed in the manuscript. This includes employment, consultancies, honoraria, stock ownership or options, expert testimony, grants or patents received or pending, or royalties.

## Ethical approval

The study protocol was approved by the Institute Ethics Committee vide letter number: IEC-601/03.09.2021, RP-24/2021.

## Availability of data and material

Data regarding this study will be available from the corresponding author (SR) on reasonable request.

## Information about writing assistance

No funded writing assistance was utilised in the production of this manuscript.

## Figures and Tables

**Table 1.** Baseline characteristics (*n* = 120).

Parameter	*N* (%)
Age at diagnosis (years) median (range)	53 (20–79)
Sex Males Females	81 (67.5%) 39 (32.5%)
ECOG PS 0 1 2 3 Not recorded	8 (6.7%) 73 (60.8%) 23 (19.2%) 7 (5.8%) 9 (7.5%)
Site of involvement Stomach Small intestine Duodenum Jejunum Ileum Peritoneal + omental Retroperitoneal Anorectal region Others	60 (50%) 42 (35%) 33.3% (14/42) 45.2% (19/42) 21.4% (9/42) 5 (4.1%) 2 (1.6%) 4 (3.3%) 7 (5.8%)
Metastatic at presentation Yes No	67 (55.8%) 53 (44.1%)

**Table 2.** AFIP criteria for risk stratification.

	Size (cm)	Mitotic rate (per 50 HPF)	Gastric	Jejunoileal	Duodenal	Rectal
1	≤2	≤5	None	None	None	None
2	2–5	≤5	Very low	Low	Low	Low
3A	5–10	≤5	Low	Moderate		
3B	>10	≤5	Moderate	High	High	High
4	≤2	>5				High
5	2–5	>5	Moderate	Moderate	High	High
6A	5–10	>5	High	High		
6B	>10	>5	High	High	High	High

**Table 3.** Details of mutational analysis (*n* = 73).

Type	N (%)	Primary site
C-kit mutation • Exon 9 • Exon 11 • Exon 13 • Exon 17	38 (52%) 7 26 1 1	Stomach: 17 Small bowel: 14 Peritoneal/omental: 3 Liver: 1
PDGFR mutation • Exon 12 • Exon 18 PDGFRA D842V	14 (19.2%) 3 10 4	Stomach: 8 Small bowel: 5 Peritoneal: 1
Wild type SDH-deficient NTRK	12 (16.4%) 8 (10.9%) 1 (1.3%)	Stomach: 7/small bowel: 4 Peritoneal: 1 Stomach: 7/duodenum: 1 Stomach: 1
Genetic syndromes • NF1 • Carney’s triad	1 (Wild type, SDH sufficient; NF1 mutation detected in exon 45) 1 (SDH deficient/mutation +ve)	

**Table 4.** Treatment changes after mutation testing.

Change of Rx plan	*N* = 29
Stopped TKI	3
Switched TKI	23
Increase dose of TKI	2
Opted for surgery versus TKI	1

**Table 5.** Two-year OS of patients with metastatic disease as per their mutation profile.

Mutation	2-year OS (%)
cKIT Exon 9	75
cKIT Exon 11	88
PDGFR Exon 18	66
SDH	66
Wild-type GIST	75

**Table 6.** The overall RRs of different TKIs used in the metastatic setting.

TKIs	Setting	Overall RRs	Adverse effects
Imatinib	First line	46.4% (26/56)	Aanemia, fatigue, oedema, and hypopigmentation
Sunitinib	Second line	13.3% (2/15)	Hypertension, diarrhoea, hand-foot syndrome
Regorafenib	Third line	28.5% (2/7)	Anaemia, diarrhoea, nausea, transaminitis
Avapritinib	Fourth line	20% (1/5)	Mood and sleep disorder Subdural haemorrhage
Ripretinib	Fourth line	100% (3/3)	Thrombocytopenia, fatigue
Cabozantinib	Fourth/fifth line	0% (0/2)	Transaminitis, hypertension, diarrhoea
